# Neuronal ablation of GHSR mitigates diet-induced depression and memory impairment via AMPK-autophagy signaling-mediated inflammation

**DOI:** 10.3389/fimmu.2024.1339937

**Published:** 2024-02-23

**Authors:** Hongying Wang, Zheng Shen, Chia-Shan Wu, Pengfei Ji, Ji Yeon Noh, Cédric G. Geoffroy, Sunja Kim, David Threadgill, Jianrong Li, Yu Zhou, Xiaoqiu Xiao, Hui Zheng, Yuxiang Sun

**Affiliations:** ^1^ Department of Nutrition, Texas A&M University, College Station, TX, United States; ^2^ Department of Endocrinology, Chongqing Key Laboratory of Translational Medicine in Major Metabolic Diseases, First Affiliated Hospital of Chongqing Medical University, Chongqing, China; ^3^ Department of Neuroscience & Experimental Therapeutics, Texas A&M University, College Station, TX, United States; ^4^ Texas A&M Institute for Genome Sciences and Society, Texas A&M University, College Station, TX, United States; ^5^ Department of Molecular and Cellular Medicine, Texas A&M University, College Station, TX, United States; ^6^ Department of Veterinary Integrative Biosciences, Texas A&M University, College Station, TX, United States; ^7^ Department of Health and Life Sciences, University of Health and Rehabilitation Sciences, Qingdao, Shandong, China; ^8^ Huffington Center on Aging, Baylor College of Medicine, Houston, TX, United States

**Keywords:** obesity, behaviors, neuroinflammation, GHSR, AMPK, autophagy

## Abstract

Obesity is associated with chronic inflammation in the central nervous system (CNS), and neuroinflammation has been shown to have detrimental effects on mood and cognition. The growth hormone secretagogue receptor (GHSR), the biologically relevant receptor of the orexigenic hormone ghrelin, is primarily expressed in the brain. Our previous study showed that neuronal GHSR deletion prevents high-fat diet-induced obesity (DIO). Here, we investigated the effect of neuronal GHSR deletion on emotional and cognitive functions in DIO. The neuron-specific GHSR-deficient mice exhibited reduced depression and improved spatial memory compared to littermate controls under DIO. We further examined the cortex and hippocampus, the major regions regulating cognitive and emotional behaviors, and found that the neuronal deletion of GHSR reduced DIO-induced neuroinflammation by suppressing proinflammatory chemokines/cytokines and decreasing microglial activation. Furthermore, our data showed that neuronal GHSR deletion suppresses neuroinflammation by downregulating AMPK-autophagy signaling in neurons. In conclusion, our data reveal that neuronal GHSR inhibition protects against DIO-induced depressive-like behavior and spatial cognitive dysfunction, at least in part, through AMPK-autophagy signaling-mediated neuroinflammation.

## 1 Introduction

Ghrelin, a 28 amino acid orexigenic hormone predominantly synthesized by the X/A-like enteroendocrine cells of the stomach (1), has a wide range of biological functions, such as regulating energy homeostasis, metabolism, inflammation, and neuronal functions (2–4). The best-known functions of ghrelin are to stimulate growth hormone (GH) secretion (5), increase appetite (6), and promote adiposity (7). Ghrelin functions by signaling through its receptor, the growth hormone secretagogue receptor (GHSR) (5, 8). In contrast to ghrelin, GHSR is primarily expressed in the brain, including the hippocampus, cerebral cortex, hypothalamus, and midbrain (9–11), and its expression in peripheral tissues is much lower (10, 11). It has been reported that global GHSR knockout attenuates diet-induced obesity (DIO) (12). We showed that GHSR regulates macrophage polarization in adipose tissue, and global GHSR deletion mitigates toxic diet-induced inflammation (13, 14). In addition, we found that global GHSR ablation alleviates age-related obesity and insulin resistance by promoting thermogenesis (15), and our collective work showed that the deletion of GHSR in Kiss1 neurons reduces DIO-associated anxiety-like behavior (16). Moreover, our previous study revealed that pan-neuronal GHSR deletion almost completely prevents DIO and shows increased sympathetic activity-associated thermogenesis (17). However, it is unknown whether neuronal GHSR affects emotional and cognitive behaviors under DIO.

Obesity is a serious health issue with increasing prevalence worldwide (18, 19), and obesity is a major risk factor for neurodegenerative diseases and psychiatric disorders (20, 21). We and others have demonstrated that mice with Western diet-induced DIO exhibit cognitive dysfunction, depression, and anxiety behaviors (22–25). Obesity is also known to be accompanied by low-grade chronic inflammation showing increased proinflammatory chemokines and cytokines, such as monocyte chemotactic protein-1 (MCP1), interleukin-1β (IL-1β), interleukin-6 (IL-6) and tumor necrosis factor-α (TNF-α) (26). Neuroinflammation occurs in the hippocampus, cortex, and amygdala of obese mice (27–29). Inflammation is a common pathological feature of many neurodegenerative conditions and behavior disorders. Neuroinflammation is controlled by resident microglia and astrocytes, as well as infiltrated immune cells in the brain; these cells work cooperatively to modulate neural activity, plasticity, and repair (30–33). Microglia, the resident myeloid cells in the brain, play a major role in immune surveillance (33). Astrocytes form functional barriers and borders that help to control the trafficking of immune cells into nonneural cellular compartments in the CNS (30). AMP-activated protein kinase (AMPK) is a major nutrient sensor that regulates cellular energy balance (34, 35). In response to low energy levels, AMPK is phosphorylated to promote ATP generation and inhibit energy consumption to maintain energy homeostasis (36). It has been reported that hypothalamic AMPK activity is significantly altered in DIO mice, indicative of an impairment of energy homeostasis under DIO (37). It has also been demonstrated that neuronal AMPK has a regulatory role in amyloidogenesis; increased AMPK phosphorylation has been observed in dementia; more importantly, genetic removal of the AMPK α2-subunit prevents amyloid β-induced long-term potentiation impairment (38, 39). Autophagy is a canonical intracellular process regulated by AMPK and mammalian target of rapamycin (mTOR), and the regulation of autophagy has been tightly linked to cognition (40–42). However, it is unclear whether neuronal GHSR has a role in regulating these signaling pathways and remodeling neuroimmune networks. The hippocampus and cortex are key regions that control emotions, learning and memory, showing pathological changes in psychiatric disorders such as depression and neurodegenerative impairments such as dementia (43–45). In this study, we investigated the effects of neuronal GHSR on psychiatric/cognitive functions and neuroinflammation pathology under DIO using a mouse model in which GHSR is selectively deleted in neurons and investigated the associated signaling pathways in relevant brain regions and neuronal cells.

## 2 Materials and methods

### 2.1 Animals

As we previously reported, the GHSR floxed mice were fully backcrossed on a C57BL/6J background. Using a Cre-Lox system, we generated neuronal-specific GHSR-deficient (*Syn1-Cre; Ghsr ^f/f^*) mice by breeding *Ghsr^f/f^* mice with widely used pan-neuronal *Synapsin 1 (Syn1)-Cre* mice (17). To identify *Syn1-Cre*-activated neurons, we assessed reporter tdTomato expression by crossing *Syn1-Cre* mice with Rosa26-tdTomato mice. To validate GHSR expression in various brain regions, we studied GHSR surrogate green fluorescence protein (GFP) expression using *GHSR – IRES - tauGFP* knock-in mice, where the GFP reporter integrated into the GHSR locus and translationally controlled by the GHSR promoter (46). Mice were housed in the animal facility of Texas A&M University (College Station, Texas) and maintained on 12-hour light and 12-hour dark cycles (lights on at 6:00 AM) at 75° F ± 1 ° F. Food and water were available *ad libitum.* For this study, age-matched male *Syn1-Cre; Ghsr ^f/f^* mice and their littermates *Ghsr^f/f^* were fed a high-fat diet (HFD) starting from 2 months of age. Diets were purchased from Harlan Teklad (Madison, WI, USA): regular diet (RD): (Cat# 2018) with a caloric composition of 18% from fat, 58% from carbohydrates, 24% from protein, and HFD: (Cat# TD88137) with a caloric composition of 42% from fat, 42.7% from carbohydrates, 15.2% from protein. After 6 months of HFD feeding, mice were subjected to a series behavioral test and a less stressful test was done first. Therefore, the chronological order of the behavioral experiments was the open field test → forced swim test → novel object recognition test → Morris water maze test; one week recover was given between tests or after test. At last, 9-10 months old mice were anesthetized with isoflurane before sacrifice for tissue collection. All animal experimental protocols were approved by the institutional animal care and use committee of Texas A&M University.

### 2.2 Open field test

The open field test was adapted from previous publications (16, 47, 48). Briefly, each mouse was allowed to stay in a standardized chamber (50 cm length x 50 cm width x 38 cm height) for 30 minutes in the Coulbourn Tru-Scan system located at the Rodent Preclinical Phenotyping Core at Texas A&M University. The mice naturally like to stay at the corner/outer zone of the square, therefore, the travel distance and time spent in the outer and inner zones were analyzed using Tru-Scan software.

### 2.3 Forced swim test

The forced swim test (FST) was performed as previously described (49). There was one 6 min-long session for each mouse, divided into a pretest (the first 2 min) and a test (the last 4 min). For acclimation, mice were transported to the testing environment at least 30 min prior to the test. Standardized transparent cylinders (50 cm in height and 20 cm in diameter) were filled with ~25 °C water, and the water depth was adjusted according to the mouse’s size to ensure that the bottom of the cylinder was not touched. Then, each mouse was placed in a water-filled cylinder for 6 min, and a camera was used to record the process. The mice were first trying to escape but eventually exhibited immobility. The immobility time was defined as the duration of time when the mouse was floating just to keep the nose above water but without any movement.

### 2.4 Novel object recognition test

Mice naturally tend to explore newer things. The recognition of mice was assessed using the new object recognition test (NORT) as we previously described (24). Mice were trained for the task for three consecutive days. On the first day, mice were submitted to a 5-min acclimatization session to the open field apparatus (long × width × high: 40 cm × 40 cm × 50 cm) made of transparent plastic sheets without objects. Twenty-four hours after the acclimatization session, two identical objects (A1 and A2) were placed in two fixed adjacent corners (approximately 9 cm from the wall); mice were allowed to explore two identical objects for 5 min. Two hours after the exploration of two identical objects, mice underwent a short-term memory test, during which they were allowed to explore the apparatus for 5 min in the presence of two objects with a new object (familiar object A1 and new object B). Twenty-four hours after the short-term memory test, the mice were returned to explore familiar object A1 and new object B for 5 min to determine long-term memory. Object exploration frequency was defined as sniffing or touching the object with the nose. A “recognition index” was calculated for each animal as expressed by the ratio TB/(TA+TB) (TA=times of exploring the familiar object; TB=times of exploring the novel object).

### 2.5 Morris water maze

The Morris water maze (MWM) was performed as we and others previously described (24, 50) to evaluate the rodent spatial learning and memory. Briefly, the water maze consisted of a standard pool of 150 cm in diameter and 70 cm in height filled with water (25 ± 1°C) made opaque with nontoxic tempera paint. White curtains surrounded the pool fixed with 3 distal visual cues, and the pool was divided into 4 quadrants (NE, NW, SW, and SE). A CCD camera and tracking system software were used to record the swim paths of the mice. The test included spatial training and a probe test. Twenty-four hours before spatial training, the mice were allowed to adapt to the pool for a 120 s free swim test. Then, the mice were trained in the spatial learning session consisting of 6 trials per day for five consecutive days. In each trial, mice were placed in the water at different positions (NE, NW, SW, SE, N, E) facing the pool wall. Then, they were required to swim to find a hidden platform (13 cm in diameter, located in the SW quadrant), which was submerged 1 cm below the water. During each trial, mice were allowed to swim until they found the hidden platform and stayed on the platform for 20 s before being returned to the holding cage. Mice that failed to find the hidden platform in 120 s were guided to the platform where they remained for 20 s. Twenty-four hours after the last trial, a probe test was performed. Mice were placed into the pool from the point of NE without the platform for 120 s, and their swimming paths were recorded. The following parameters were analyzed: time to the platform, time spent in the target quadrant, number of platform crossings, swimming speed, and distance traveled.

### 2.6 Quantitative real-time PCR

Total RNA was isolated using the Aurum™ Total RNA Mini Kit (*Bio-Rad*) following the manufacturer’s instructions. The complementary DNA was synthesized from 500 ng RNA using the iScript™ Reverse Transcription Supermix (*Bio-Rad*). Quantitative real-time PCR was performed in duplicate using SsoAdvanced™ Universal SYBR^®^ Green Supermix (*Bio-Rad*) and then detected on a CFX384 Touch™ Real-Time PCR Detection System (*Bio-Rad*). Relative mRNA expression levels were normalized to the housekeeping gene 18S. The GHSR-1a primers were forward primer 5’-GGACCAGAACCACAAACAGACA-3’ and reverse primer 5’-CAGCAGAGGATGAAAGCAAACA-3’ (10). Information for other primers is available upon request. The expression levels of various genes in the *Syn1-cre; Ghsr^f/f^* group were normalized to that of *Ghsr^f/f^* group.

### 2.7 Western blotting

Frozen mouse tissues, including the hippocampus and cortex, and N2A cells were homogenized in RIPA buffer with Complete Protease Inhibitor Cocktail from Roche (Cat# 04693159001) and PhosSTOP phosphatase inhibitor cocktail from Roche (Cat #4906837001). The concentration of total protein was determined using a BCA protein assay kit (Cat # 23225, Thermo, Pierce). Aliquots of 60 µg protein from each sample were separated by sodium dodecyl sulfate-polyacrylamide gel electrophoresis (SDS-PAGE) and then transferred to nitrocellulose membranes for immunoblot analysis. The following antibodies purchased from Cell Signaling Technologies were used: anti-GAPDH (CST, #2118), anti-GFAP (CST, #3670), anti-beclin1 (CST, #3495), anti-SQSTM1/p62 (CST, #5114), anti- LC3A/B (CST, #4108), anti-p-AMPKα (Thr172) (CST, #2535), anti-AMPK (CST, #2603), anti-p-mTOR (ser2448) (CST, #2971), and anti-mTOR (CST, #2972), anti-β-actin (CST, #4967), anti-ATG7 (CST, #8558). The antibodies were diluted in a 1:1000 ratio in tris buffered saline with 0.1% Tween-20 and 5% bovine serum albumin (BSA). SignalFire ECL Reagent (Cell Signaling, #6883) was used for detection in the Western blotting analysis. The quantitation was normalized by housekeeping GAPDH or beta-actin, and the phosphorylated protein levels were normalized by their perspective total protein levels.

### 2.8 Immunofluorescence microscopy

The brains were removed and fixed in 10% formalin for 24 hrs. Later, the brains were cryoprotected by immersion in 15% sucrose in PBS and then 30% sucrose in PBS at 4 °C until sectioning. Coronal sections (40 µm) were cut via a vibratome (LEICA VT1000 S). Staining was performed on floating sections. The slices were immunostained with antibodies (anti-GFAP, Millipore, AB5541; anti-NeuN, Abcam, ab104224) at a 1:500 dilution, mounted onto super frosted glass slides and cover-slipped with Mounting Shield with DAPI (Vector Laboratories, Burlingame, CA, USA). Images were taken with a Leica confocal microscope (LEICA TCS SPE).

### 2.9 RNA *in situ* hybridization

*Ghsr* and *Rbfox3* mRNA were co-stained by the commercial RNAscope^®^ 2.5 HD Duplex Detection Kit (ACD, Cat #322500). RNAscope™ Probe- Mm-Ghsr-O2-C1 (ACD, Cat #1147851-C1) and RNAscope^®^ Probe- Mm-Rbfox3-C2 (ACD, Cat #313311-C2) were used. All procedures strictly followed the manufacturer’s instructions.

### 2.10 Neuro-2A cell line culture and *in vitro* study

The Neuro-2A (N2A) cell line was purchased from ATCC (Manassas, VA, USA) and grown in Corning™ Eagles’ Minimum Essential Medium (MEM) with 10% fetal bovine serum (FBS) and 1% penicillin streptomycin (P/S). For the *in vitro* study, N2A cells were seeded onto 6-well treated plates at a density of 1.0 x 10^6^ cells per well and incubated at 37°C and 5% CO2 overnight. To inhibit GHSR expression and autophagy in N2A cells, siRNA-*Ghsr* (siR-*Ghsr*, 100 pmol/ml) and siRNA-*Atg7* (siR-*Atg7*, 100 pmol/ml) were transfected into N2A cells with Lipofectamine 3000 following the manufacturer’s instructions (Life Technologies Corporation, Carlsbad, CA, USA). N2A cells in the control groups were transfected with siRNA-Scramble (Scramble, 100 pmol/ml) (Life Technologies Corporation, Carlsbad, CA, USA). After 16 hours of transfection, the cell culture medium was replaced with MEM fasting medium (MEM with 1% FBS and 1% P/S). In palmitate-treated groups, BSA-palmitate saturated fatty acid complexes (PAL, final concentration was 100 µM, in which BSA final concentration was 16 µM.) (Cayman Chemical, Ann Arbor, MI, USA) were used. In the corresponding control groups, BSA control (final concentration was 16 µM) for BSA-fatty acid complexes (Cayman Chemical (Ann Arbor, MI, USA) was used. After 24 hours of PAL treatment, nucleic acid and protein samples of the cells were collected for RT-qPCR and western blotting assays, respectively.

### 2.11 Statistical analysis

Numeric data are presented as the mean ± SEM. A two-tailed *t* test and one or two-way analysis of variance (ANOVA) with Tukey’s *post hoc* test or Tukey’s multiple comparisons test were used. A *P* value less than 0.05 was considered statistically significant.

## 3 Results

### 3.1 Synapsin1-specific Cre activates GHSR expression in brain regions relevant to cognitive functions

**Figure 1:**
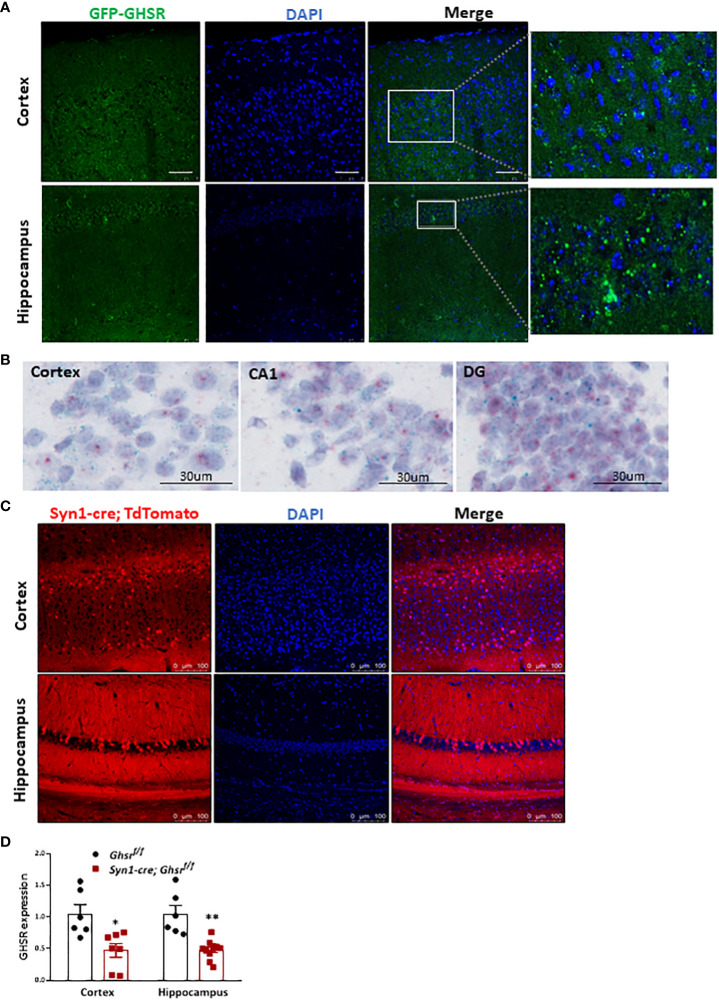
Syn1-cre activated neurons and GHSR expression in the normal brain. **(A)** GHSR expression in the cortex and hippocampus in GHSR-IRES-tauGFP mice. Scale bar, 50 µm, under a 20x objective. **(B)** Representative images of RNA *in situ* hybridization in the adult mouse cortex (CTX), and hippocampus including CA1 and dental gyrus (DG). The bluish green dots represented *Ghsr* mRNA expression; The red dots represented *Rbfox3* (Neuron marker) mRNA expression; The purple color presented DNA counterstained with hematoxylin. Under a 40x objective; scale bar, 30 µm. **(C)** Coronal section showing Syn1-cre-activated tdTomato-positive neurons (red fluorescence) in the cortex and hippocampus. Scale bar, 100 µm, under a 20x objective. **(D)** GHSR mRNA gene expression in the cortex and hippocampus by quantitative real-time PCR (Mouse number n=6-10). *p<0.05, **p<0.01, *Ghsr ^f/f^* vs. *Syn1-cre; Ghsr ^f/f^*. All data are presented as the means ± SEMs.

Studies have shown that there is no good GHSR antibody that is truly specific for mice, so we used GFP as a surrogate of GHSR to study GHSR expression in the brain. We used *GHSR-IRES-tauGFP* mice in which the GFP reporter was integrated into the GHSR locus and translationally controlled by the GHSR promoter (46). Specifically, related to cognitive function, we detected ample GFP expression in the cortex and hippocampus, which indicates that GHSR is highly expressed in these cognition-relevant regions (**Figure 1A**). We also used *in situ* hybridization RNAscope probes to detect *Ghsr* mRNA (bluish green tiny dots) and neuron marker *Rbfox3* mRNA (tiny red dots) in the cortex (CTX), and hippocampus including CA1 and dental gyrus (DG) (**Figure 1B**), confirming that *Ghsr* is highly expressed in the neurons. To directly visualize Synapsin1 (*Syn1)*-specific Cre-activated cells following Cre-mediated recombination, we crossed *Syn1-Cre* mice with *Rosa26-tdTomato* reporter mice, which express red fluorescent protein. *Syn1-Cre* activated red tdTomato fluorescence was evident in the cortex and hippocampus regions (**Figure 1C**). We previously validated that GHSR deletion in *Syn1-Cre; Ghsr ^f/f^* mice was restricted to the brain and no other peripheral tissues (17). Here, we further verified GHSR mRNA expression in the cortex and hippocampus. In the cortex and hippocampus of *Syn1-Cre; Ghsr ^f/f^* mice, we detected more than 60% reduced GHSR expression compared to that of *Ghsr ^f/f^* mice, indicating that GHSR deletion is effective in neuronal regions relevant to cognition (**Figure 1D**).

### 3.2 Neuronal deletion of GHSR attenuates DIO-associated spatial memory impairment

**Figure 2:**
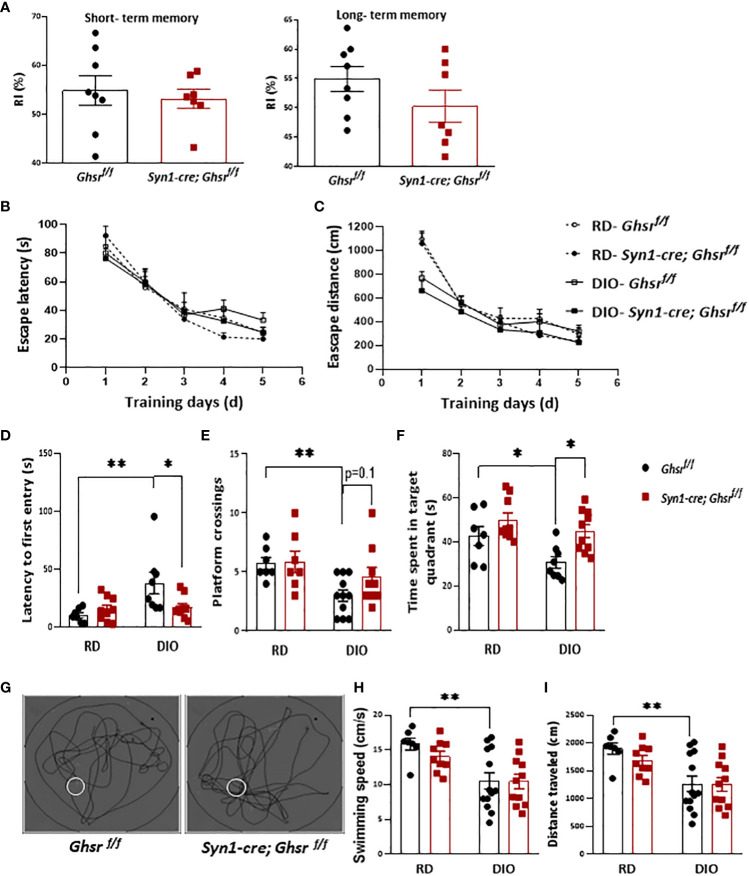
*Ghsr ^f/f^* mice and *Syn1-cre; Ghsr ^f/f^* mice were subjected to learning and memory behavior tests. **(A)** Novel object recognition test to assess short- and long-term memory under high-fat diet-induced obesity (DIO). RI: recognition index. **(B, C)** Escape latency and distance during training days of MWM under regular diet (RD) and DIO. **(D–F)** The time spent locating the platform for the first time, the number of platform crossings, and the time spent in the platform located quadrant during the probe test of the MWM in RD and DIO mice. **(G)** Representative swim paths during the probe test under DIO; the white circle represents the platform. **(H, I)** The swimming speeds and distance traveled during the probe test of the MWM under RD and DIO. Mouse number n= 7-13. The mice were 9-10 months of age. *p<0.05, **P<0.01. All data are presented as the means ± SEMs.

As obesity is associated with cognitive dysfunction, including memory, and learning impairments (24, 25), we assessed whether neuronal loss of GHSR affects DIO-induced impairment in learning and memory. The novel object recognition test showed that there was no difference in short- and long-term memory between the genotypes under DIO (**Figure 2A**). We further subjected the mice to the Morris water maze (MWM) to evaluate spatial learning and memory. During the training phase, there was no significant difference between either the diets or genotypes in terms of the escape latency and travel distance (**Figures 2B, C**). During the probe test (on the last day of the test), as expected, control *Ghsr ^f/f^* mice under DIO showed spatial memory deficits compared to *Ghsr ^f/f^* mice under RD feeding, taking more time to find the platform location the first time and spending less time in the target quadrant where the platform was located and fewer platform crossings (**Figures 2D–F**). Interestingly, we found that *Syn1-Cre; Ghsr ^f/f^* mice spent less time to find the platform for the first time, spent more time in the target quadrant, and showed a trend of more platform crossings during the probe test (**Figures 2D–G**), suggesting that *Syn1-Cre; Ghsr^f/f^* mice have better spatial memory. While the HFD-fed mice showed a much slower swimming speed and shorter travel distance, there was no difference between genotypes (**Figures 2H, I**), which suggests that the reduced body weight of HFD-fed *Syn1-Cre; Ghsr ^f/f^* mice did not accelerate swimming speed and travel distance in behavioral tests. Collectively, these results indicated that neuron-specific deletion of GHSR significantly attenuated DIO-associated spatial memory impairment.

### 3.3 Neuronal deletion of GHSR mitigates DIO-induced depression but has no effect on anxiety

**Figure 3:**
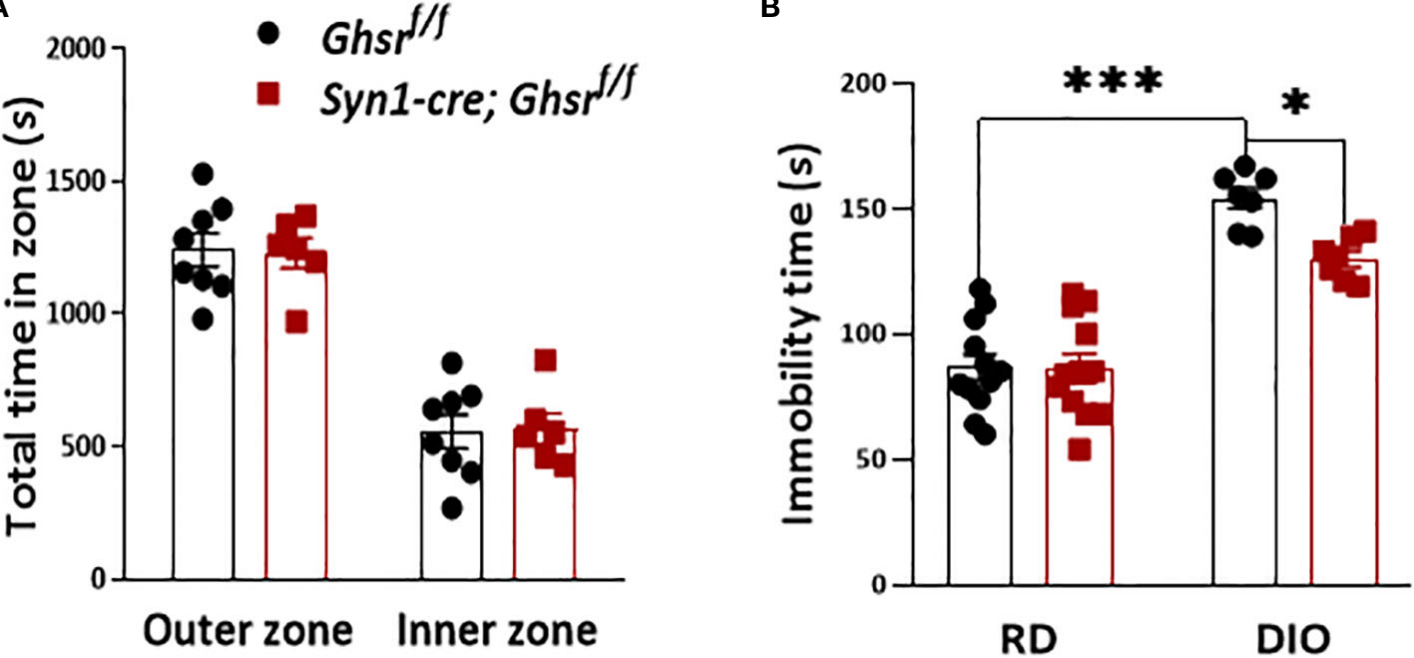
*Ghsr ^f/f^* mice and *Syn1-cre; Ghsr ^f/f^* mice with DIO were subjected to behavioral tests of anxiety and depression. **(A)** Open field test: 8-month-old mice were allowed to explore the chamber for 30 min. The time spent in different zones was recorded during the test. **(B)** Under RD and DIO, forced swim test: 8-month-old mice were acclimated to the water for 2 min, and immobility time was recorded for an additional 4 min. Mouse number n= 6-12. *p<0.05, ***p<0.001. All data are presented as the means ± SEMs.

Obesity is also associated with neuropsychiatric disorders, including anxiety and depression (21, 23). To evaluate the anxiety state of *Syn1-Cre; Ghsr ^f/f^* mice with DIO, the mice were subjected to an open field test. We found that there was no difference in the basal anxiety parameters between genotypes (**Figure 3A**). The forced swimming test (FST) was used to assess depression-related behavior, in which immobility time was used as a readout of behavioral despair (49). Similar to previous studies (22, 23, 25), we found that DIO mice exhibited a depressive-like state by showing significantly more immobility time than RD-fed mice, while HFD-fed *Syn1-cre; Ghsr ^f/f^* mice had a significantly less immobility time than HFD-fed *Ghsr ^f/f^* mice in the FST (**Figure 3B**), suggesting that neuronal deletion of GHSR protects against DIO-induced depression.

### 3.4 Neuronal deletion of GHSR decreases DIO-induced neuroinflammation in the cortex and hippocampus

**Figure 4:**
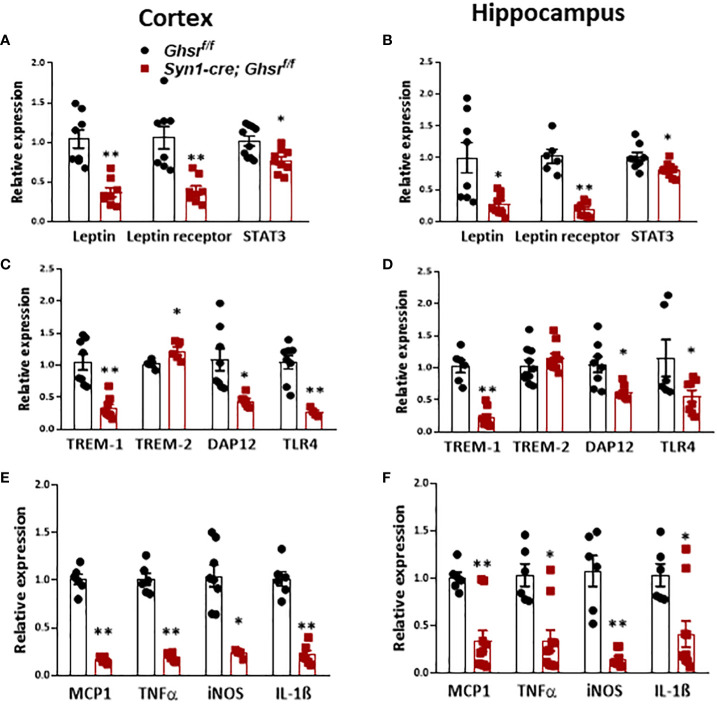
Proinflammatory cytokines in the cortex and hippocampus of mice with DIO. Mice were sacrificed at the age of 10 months (HFD started at the age of 2 months). **(A, B)** Gene expression of leptin, leptin receptor, and STAT3 in the cortex and hippocampus. **(C, D)** Gene expression of TREM-1, TREM-2, DAP12, and TLR4 in the cortex and hippocampus. **(E, F)** Gene expression of inflammatory counterparts in the cortex and hippocampus. Mouse number n=4-8 in each group. *p<0.05, **p<0.01, *Ghsr ^f/f^* vs. *Syn1-cre; Ghsr ^f/f^*. All data are presented as the means ± SEMs.

Obesity is associated with low-grade chronic inflammation (26); previously, we reported that global ablation of GHSR mitigates high fructose corn syrup-induced adipose and hepatic inflammation (13). Leptin, as a proinflammatory adipokine, is known to be associated with obesity-related inflammation (51). In HFD-fed *Syn1-cre; Ghsr ^f/f^* mice, leptin and leptin receptors, along with their downstream mediator STAT3, were decreased in the cortex and hippocampus (**Figures 4A, B**). Triggering receptors expressed on myeloid cells (TREMs) are known to play pivotal roles in innate immunomodulation, and TREM-1 stimulates proinflammatory responses through the activation of proinflammatory cytokines, such as TNFα and IL-1β (52, 53). Interestingly, we found that TREM-1 expression in the cortex and hippocampus in *Syn1-Cre; Ghsr ^f/f^* mice was significantly decreased compared to that in *Ghsr ^f/f^* mice with DIO (**Figures 4C, D**). In contrast to TREM-1, TREM-2 in microglia has been reported to have important protective functions, such as phagocytosis of dead neurons, inflammation control and tissue repair (54). Indeed, we found that TREM-2 expression was significantly higher in the cortex and showed a trend of increase in the hippocampus of HFD-fed *Syn1-Cre; Ghsr ^f/f^* mice (**Figures 4C, D**). In addition, we also assessed the downstream mediator DAP12 of TREM signaling. Consistently, DAP12 expression in the cortex and hippocampus of *Syn1-Cre; Ghsr ^f/f^* mice was markedly reduced (**Figures 4C, D**). It has been reported that TREM-1 amplifies inflammation induced by Toll-like receptors (TLRs) (55); indeed, we detected a decrease in TLR4 in the cortex and hippocampus of HFD-fed *Syn1-Cre; Ghsr ^f/f^* mice (**Figures 4C, D**). Furthermore, the proinflammatory chemokine/cytokine expression of MCP1, TNFα, iNOS, and IL-1β in the cortex and hippocampus of HFD-fed *Syn1-Cre; Ghsr ^f/f^* mice was also decreased (**Figures 4E, F**). Taken together, these results strongly support that the neuronal deletion of GHSR reduces DIO-induced neuroinflammation in the cortex and hippocampus and influences various inflammation-associated signaling pathways.

### 3.5 Neuronal deletion of GHSR increases GFAP reactivity and decreases microgliosis in the cortex and hippocampus under DIO

**Figure 5:**
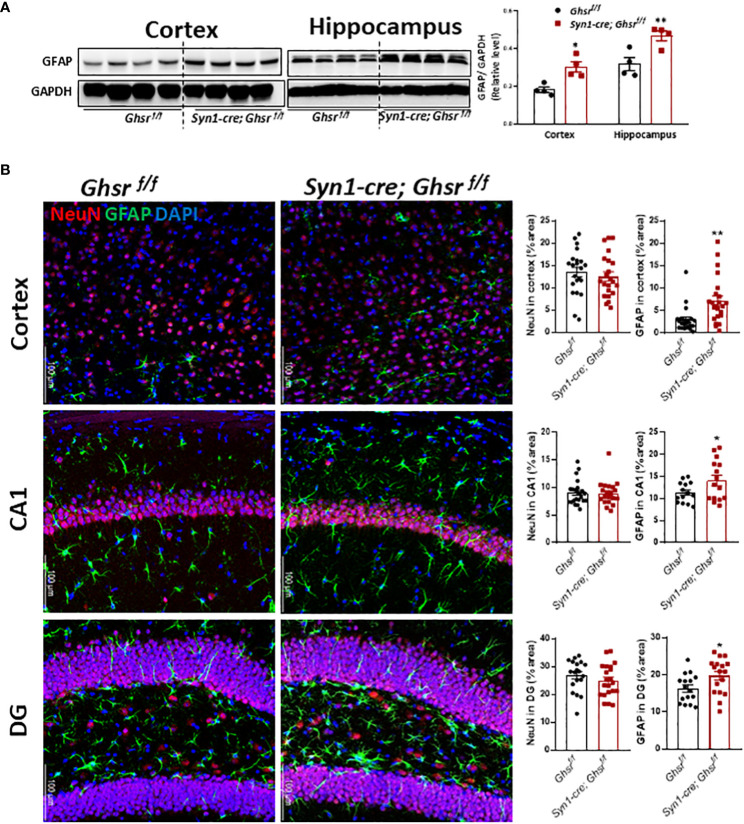
Characterization of astrocytes. The HFD was started at 2 months of age, and all mice were sacrificed at 9-10 months of age. **(A)** Representative immunoblots of GFAP expression in the cortex and hippocampus and relative quantitation (Mouse number n=4). **(B)** Representative coronal sections of immunofluorescence and area quantification in the regions of cortes, hippocampi CA1 and dentate gyrus (DG). NeuN-labeled neurons (red), GFAP-labeled astrocytes (green), and nuclear counterstain (DAPI, blue) (Mouse number n= 6-7, 3-4 sections/mouse brain). Scale bar 100 µm. Under a 20x objective. *p<0.05, **p<0.01, *Ghsr ^f/f^* mice vs. *Syn1-cre; Ghsr ^f/f^*. All data are presented as the means ± SEMs.

**Figure 6:**
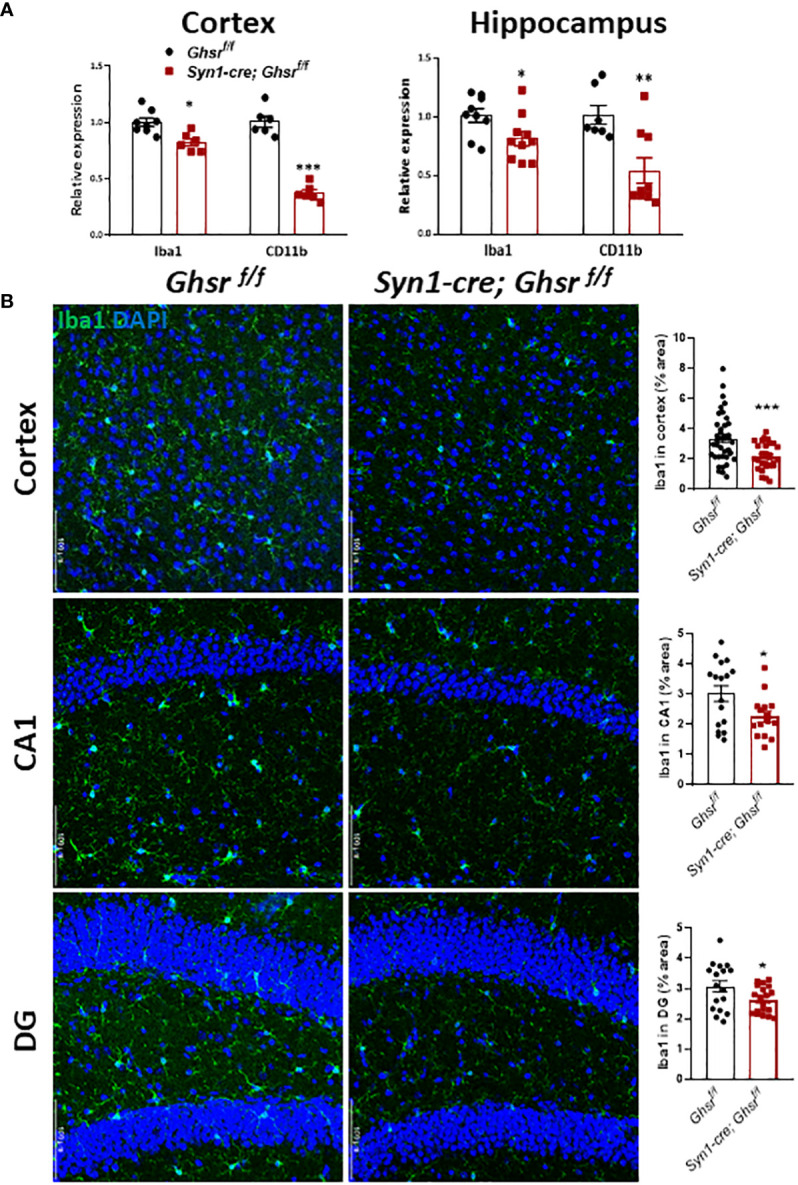
Characterization of microglia. The HFD was started at 2 months of age, and all mice were sacrificed at 9-10 months of age. **(A)** Gene expression of Iba1 and CD11b in the cortex and hippocampus, Mouse number n=6-9. **(B)** Representative coronal sections of immunofluorescence and area quantification of the cortex, hippocampi CA1 and DG regions. Iba1-labeled microglia (green), and nuclear counterstain (DAPI, blue) (Mouse number n= 6-7, 3-4 sections/mouse brain). Scale bar 50 µm. Under a 20x objective. *p<0.05, **p<0.01, ***p<0.001, *Ghsr ^f/f^* vs. *Syn1-cre; Ghsr ^f/f^*. All data are presented as the means ± SEMs.

To decipher the cell types underpinning GHSR-mediated neuroinflammation, we next examined glial cell populations in the brain. We assessed the active astrocyte marker GFAP (glial fibrillary acidic protein) by immunoblotting and immunofluorescence staining and found that GFAP protein expression was markedly increased in the cortex and hippocampus of *Syn1-Cre; Ghsr ^f/f^* mice, while the neuronal marker NeuN remained unchanged between genotypes (**Figures 5A, B**). Neuroinflammation is mainly regulated by microglia, so we measured the expression of the microglial marker Iba1 (ionized calcium-binding adapter molecule 1) and the cell surface marker CD11b in the cortex and hippocampus. The gene expression of Iba1 and CD11b in *Syn1-Cre; Ghsr ^f/f^* mice was significantly reduced in both the cortex and hippocampus (**Figure 6A**). Consistently, analysis of immunofluorescence staining showed that the number of Iba1+ microglia was reduced in the cortex and hippocampus of *Syn1-Cre; Ghsr ^f/f^* mice compared with littermate controls. Remarkably, Iba1+ cells in HFD-fed *Syn1-Cre; Ghsr ^f/f^* mice were less dense and less bright than those in HFD-fed Ghsr *^f/f^* mice (**Figure 6B**). Together, these results strongly support that neuronal GHSR ablation mitigates DIO-induced neuroinflammation.

Since the effect of the neuronal GHSR deletion on DIO-induced neuroinflammation was robust in the cortex and hippocampus, we sought to determine whether pathology of the suppressed inflammation also presents in other brain regions. We studied the hypothalamus, where GHSR is highly expressed. The gene expression levels of GFAP, Iba1, and CD11b in the hypothalamus were comparable between the genotypes (**Supplementary Figures A–C**). The protein levels of GFAP and Iba1 in the hypothalamus also did not change (**Supplementary Figures D, E**). These results indicate that GHSR differentially modulates glial cell activation in different brain regions under DIO and that neuronal GHSR significantly impacts glial cells in the cortex and hippocampus.

### 3.6 Neuronal deletion of GHSR reprograms AMPK-autophagy signaling in the cortex and hippocampus under DIO

**Figure 7:**
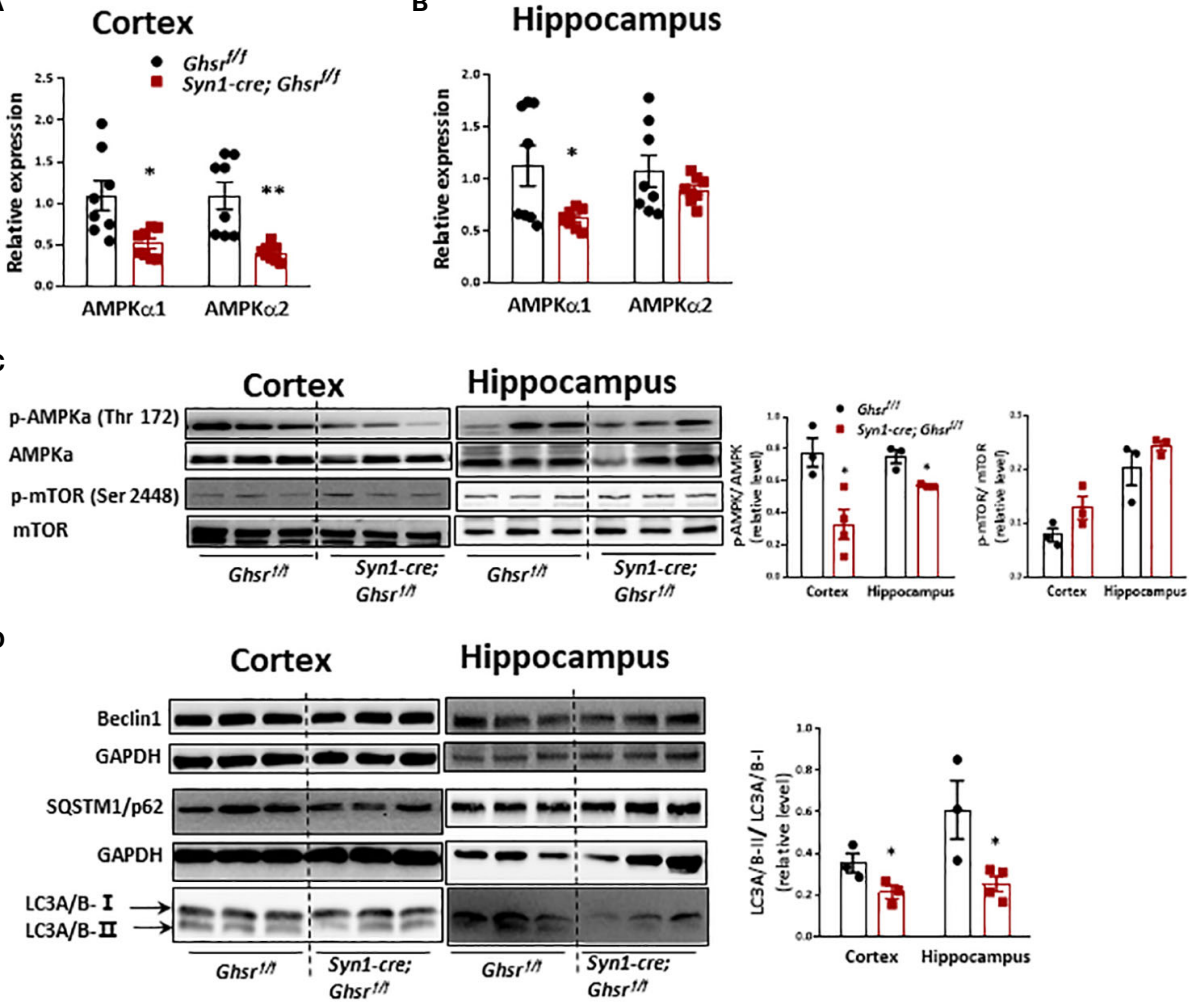
AMPK-mTOR-autophagy signaling in the cortex and hippocampus in DIO mice. The HFD was started at 2 months of age, and all mice were sacrificed at 9-10 months of age. Gene expression of AMPKa1 and AMPKa2 in the cortex **(A)** and hippocampus **(B)**. **(C)** Representative immunoblots of protein expression and corresponding quantification. **(D)** Representative immunoblots and quantification of protein expression in the cortex and hippocampus. Mouse number n=3-8, *p<0.05, **p<0.01, *Ghsr^f/f^* vs. *Syn1-cre; Ghsr^f/f^*. All data are presented as the means ± SEMs.

We are interested in determining whether neuronal deletion of GHSR ameliorates DIO-induced neuroinflammation by reprogramming AMPK-mediated signaling pathways (34). We found that cortical AMPKα1/AMPK α2 and hippocampal AMPKα1 gene expression was significantly decreased in HFD-fed *Syn1-Cre; Ghsr ^f/f^* mice (**Figures 7A, B**). In addition, there was a significant decrease in phosphorylated AMPKα (p-AMPKα) in the cortex and hippocampus of HFD-fed *Syn1-Cre; Ghsr ^f/f^* mice (**Figure 7C**). In contrast, another nutrient sensor, mTOR, was not affected in our *Syn1-Cre; Ghsr ^f/f^* mice (**Figure 7C**). Considering that autophagy is a canonical intracellular process regulated by AMPK and mTOR and that the regulation of autophagy has been tightly linked to cognition (40–42), we next evaluated neuronal autophagy activity in our *Syn1-Cre; Ghsr ^f/f^* mice. We found that the LC3A/B-II/I ratio, a well-documented marker of autophagy flux, was significantly decreased in *Syn1-Cre; Ghsr ^f/f^* mice compared to *Ghsr ^f/f^* mice with DIO (**Figure 7D**). Together, these results indicated that AMPK-autophagy signaling is reprogrammed in our *Syn1-Cre; Ghsr ^f/f^* mice.

### 3.7 Inhibition of *Ghsr* suppresses the palmitate-induced inflammatory response in N2A cells by reprogramming the AMPK-autophagy pathway

**Figure 8:**
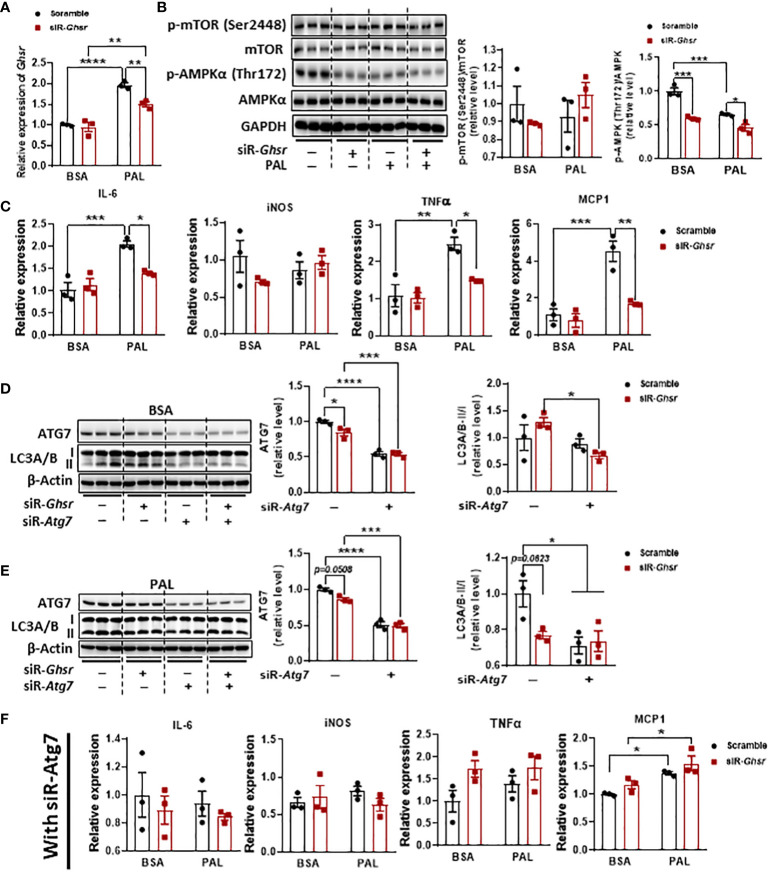
GHSR inhibition suppressed PAL-induced inflammation in N2A cells by inhibiting autophagy. **(A)** The mRNA expression of *Ghsr* in N2A cells with or without siRNA-*Ghsr* (siR-*Ghsr*) transfection under either BSA or PAL treatment. **(B)** Representative immunoblot images of p-AMPKα, AMPKα, p-mTOR, mTOR, and GAPDH proteins in N2A cells with or without siR-*Ghsr* under either BSA or PAL treatment and their corresponding quantification. **(C)** The mRNA expression of proinflammatory genes in N2A cells with or without siR-*Ghsr* under either BSA or PAL treatment. **(D, E)** Transfection of siRNA-*Atg7* (siR-*Atg7)* significantly suppresses autophagy in N2A cells under either BSA **(D)** or PAL **(E)** treatment. **(F)** Upon inhibition of autophagy, the mRNA expression of pro-inflammatory genes in N2A cells with or without siR-*Ghsr*, under either BSA or PAL treatment. PAL: BSA-conjugated palmitate, 100 µM, 24 h; siRNA concentration during transfection was 100 pmol/ml. n= 3. Data are presented as the mean ± SEM, *p<0.05, **p<0.01, ***p<0.001, ****p<0.0001.

To further confirm the roles of AMPK and autophagy in neuronal GHSR deficiency and verify that the phenotype is not body weight dependent, we investigated the direct association between GHSR and autophagy *in vitro* by using mouse neuroblast Neuro-2A (N2A) cells. N2A was exposed to palmitate to mimic a high-fat environment (56, 57). Notably, palmitate significantly suppressed AMPK phosphorylation in the saline control, confirming that palmitate induces dysregulation of intracellular energy homeostasis (**Figure 8B**). Interestingly, when we knocked down *Ghsr* in N2A cells with siRNA-*Ghsr* transfection (**Figure 8A**), the basal AMPK activity before palmitate exposure was reduced (**Figure 8B**). This reduced AMPK phosphorylation indicates that there is GHSR inhibition-mediated reprogramming of the cellular energy balance in neurons under basal conditions. With the help of such reprogramming of AMPK phosphorylation by inhibiting GHSR, the suppressive effect of palmitate on AMPK phosphorylation was no longer significant (**Figure 8B**, black bars, with siR-*Ghsr*), suggesting that the negative effects of palmitate on neuronal energy homeostasis were mitigated by GHSR inhibition. To investigate whether this GHSR inhibition-mediated AMPK reprogramming suppresses neuroinflammation, we further evaluated the genes encoding proinflammatory markers (e.g., IL-6, iNOS, TNF-α, and MCP1). Indeed, inhibition of GHSR significantly alleviated palmitate-induced proinflammatory cytokine and chemokine expression in N2A cells (**Figure 8C**).

To further confirm that autophagy is a key player contributing to GHSR deletion-mediated anti-inflammation in neurons, we inhibited autophagy activity by transfecting siRNA-*Atg7* along with siRNA-*Ghsr* before palmitate exposure (**Figures 8D, E**). Remarkably, upon inhibition of autophagy, the effect of palmitate on proinflammatory gene expression was significantly diminished (**Figure 8F**), indicating that inhibition of autophagy ameliorates the palmitate-induced inflammatory response in neurons. More importantly, the data reveals that the GHSR inhibition-induced anti-inflammatory effect was abolished when autophagy was blocked, indicating that autophagy is required for GHSR-mediated inflammatory activation in neurons. Collectively, our *in vivo* and *in vitro* results unequivocally demonstrated that inhibition of GHSR mitigates neuroinflammation under obesity, likely by reprogramming the AMPK-autophagy axis in neurons.

## 4 Discussion

As a nutrient sensor, ghrelin increases appetite and promotes obesity by signaling through its receptor GHSR (5, 7, 8). We previously reported that GHSR deficiency in neurons completely prevents DIO (17). In the current study, we induced DIO by 30 weeks of HFD feeding in *Syn1-Cre; Ghsr^f/f^* mice and found that neuronal ablation of GHSR attenuated depressive-like behavior and enhanced spatial memory. There were no behavioral differences between genotypes in RD-fed mice in depression, anxiety or cognitive tests. Thus, the GHSR-associated detrimental effect on behavior is only aggravated under DIO, not RD, suggesting that GHSR serves as a lipid-toxicity sensor in neurons. Obesity is often accompanied by neuroinflammation, which is a pathogenic trigger for behavioral dysfunctions. Indeed, neuronal ablation of GHSR in mice with DIO exhibited decreased inflammation in the cortex and hippocampus, which is in line with the improved behavioral phenotype. Interestingly, we observed that neuronal GHSR affects inflammatory markers in the cortex and hippocampus but not in the hypothalamus, which indicates that neuronal GHSR differentially regulates neuroinflammation in a region-dependent manner and that the regions of behavior are more vulnerable. Region specificity has been observed by others: 1 week of exposure to a HFD and sugar diet selectively impairs hippocampus-dependent spatial memory but not perirhinal cortex-related memory (28), and 16 weeks of HFD feeding increases inflammation in the cerebellum but not in the cortex (29).

The newly discovered improved behavioral phenotype of HFD-fed *Syn1-Cre; Ghsr^f/f^* mice is very exciting. Since HFD-fed *Syn1-Cre; Ghsr^f/f^* mice have reduced body weight, the question is whether the phenotype observed was solely due to body weight differences. While weight would have a significant impact on inflammation and neurobehaviors, we believe that differences in body weights cannot fully explain the genotype differences we observed. For example, the swimming speed and total distance traveled during the tests did not differ between *Syn1-Cre; Ghsr ^f/f^* mice and control littermates, and we believe that the improved spatial memory of neuronal GHSR-deficient mice is not fully determined by body weight differences. Additionally, we detected a cell-autonomous effect of GHSR in a neuronal cell line and observed that GHSR inhibition alters metabolic AMPK signaling under palmitate treatment, which strongly suggests that at least some of the effects of GHSR on neurons are independent of body weight. AMPK is highly expressed in neurons; AMPK activation (phosphorylation of AMPK at site Thr172) is increased in the cortex under DIO and then decreased after exercise, which is associated with reduced cortical BACE1 content, a hallmark of Alzheimer’s disease (AD) (34, 58). Our present results showed a significant decrease in AMPK activation both *in vivo* and *in vitro* with neuronal GHSR deficiency. AMPK signaling inhibition abolishes the amyloid β-induced inhibition of long-term potentiation and enhances long-term depression, and genetic deletion of the AMPKα2-subunit prevents amyloid β−induced long-term potentiation failure (38). Our data showed that neuronal-specific GHSR deletion decreases AMPK signaling in DIO mice, which suggests that GHSR might be linked to memory functions. The rapamycin target mTOR, inhibited by AMPK activation, has been recognized as an important regulator of autophagy; AMPK/mTOR signaling pathways are nutrient sensors that regulate cellular homeostasis, and dysfunction of these signaling pathways has been linked to neuronal cell death (59, 60). In addition, autophagy dysfunction has been shown to promote the onset and development of metabolic disorders, and autophagy can be either enhanced or suppressed in obesity due to dyslipidemia or overnutrition (61). Furthermore, a study has shown that autophagosomes accumulate (increased LC3-II levels) in neurons under H_2_O_2_-induced oxidative stress in AD mice (62) and that the autophagy pathway is linked to proinflammatory signaling through an increase in oxidative stress (63). In our study, neuronal deletion of GHSR in mice decreased LC3-II and iNOS in the cortex and hippocampus. More importantly, after inhibiting the autophagy pathway, the anti-inflammatory effect associated with neuronal GHSR deficiency disappears in neurons, which suggests that neuronal GHSR regulates neuroinflammation under DIO by autophagy signaling. Therefore, it is possible that GHSR deletion in neurons alters pathways, such as AMPK and autophagy, to reduce neuronal chemokine and cytokine release, which in turn reduces neuroinflammation and improves the neuron-glial network, ultimately mitigating DIO-induced behavioral impairments.

Obesity, neurodegenerative and psychiatric disorders share the common pathophysiology of chronic inflammation (29). Microglia, brain-resident macrophages, are known to play a pivotal role in the maintenance of CNS homeostasis (33, 64). In response to metabolic and/or inflammatory stresses, microglia are rapidly activated and migrate into the injury site, demonstrating a unique activated form of morphology (65). As expected, the immunoreactivity intensity of Iba1+ microglia were denser and brighter in the littermate control group, while neuronal GHSR deletion resulted in less dense and weaker Iba1 signals, indicative of reduced immunoreactivity. DIO has been shown to increase cortical and hippocampal pro-inflammatory cytokines and impair hippocampus-dependent spatial memory (28, 29). Remarkably, our neuron-specific GHSR-deleted mice with DIO showed downregulated inflammatory responses of IL-1β and leptin signaling, exhibiting improved depression in the forced swim test and better spatial memory in the water maze, which are similar to reports by others that elevated IL-1β and leptin levels in the CNS promote cognition and mood dysfunction (22, 51, 66, 67). We have reported that global ablation of GHSR mitigates depressive-like behaviors after chronic social defeat stress (47). These results together suggest that GHSR plays a pivotal role in depression and cognition under obesity, which solidifies the notion that neural GHSR is a critical link between metabolism and neurobehaviors at both the whole body and molecular levels. Under lipid insult, such as HFD intake, GHSR reprograms the metabolic pathway to induce neural injury and release chemokines, which subsequently activate the immune response in glial cells. Microglia are known to actively survey their surrounding environment and *change* their morphology and cytokine section in response to *neural* injury (68, 69), which is understandable because we detected impressive changes in glial cells of HFD-fed *Syn1-Cre; Ghsr ^f/f^* mice.

It is well documented that obesity, insulin resistance and type 2 diabetes are major risk factors for AD development, the most common form of dementia (70–72). The microglia receptor TREM2 has been shown to mitigate central inflammation and cognitive impairment in AD mice by decreasing M1-like microglia and regulating the PI3K/AKT/FoxO3a signaling pathway (43). Loss of TREM2 impairs neuronal synapses in mice (73), exacerbates cognitive impairments and reduces the microglial barrier around plaques in correlation with the decreased plaque clearance rate in AD mice (74, 75). We recently reported that overexpression of TREM2 in the hippocampus alleviates DIO-induced cognitive dysfunction and modulates microglial polarization to an anti-inflammatory state in mice, suggesting that TREM2 might be a novel target of DIO-associated cognitive impairment (76). In *Syn1-Cre; Ghsr ^f/f^* mice, the expression of the TREM family was also altered in the cortical and hippocampal regions, suggesting that TREMs might be a key mediator of GHSR-associated neuroinflammation, which can be verified in a microglial-deficient GHSR mouse model. Deleting neuronal GHSR improves depression and cognitive function and attenuates neuroinflammation in the context and hippocampus, and we believe that neuroinflammation is one of the driving forces of these behavioral phenotypes. For future studies, it would be informative to determine the role of neuronal GHSR in the modulation of learning and memory in AD models. GHSR is also expressed in astrocytes, which mediates ghrelin-induced astrocytoma cell motility (77, 78). A study has shown that ghrelin affects neurons and endothelial cells in neurodegenerative diseases or injury by dumping the activation of astroglia and microglia by reducing the excess release of proinflammatory factors (77). It has been reported that astrocytes are necessary for neuronal plasticity and memory enhancement and activated astrocytes in the hippocampus have been shown to enhance synaptic potentiation and improve cognitive performance (79). Interestingly, our HFD-fed *Syn1-Cre; Ghsr ^f/f^* mice showed remarkably more astrocytes than control *Ghsr ^f/f^* mice in the cortex and hippocampus, and the mice showed improved cognition accordingly. Collectively, our data revealed the differential intensity of interactions between neurons and the glial cell network under DIO, and neuronal GHSR serves as a critical modulator of neuroinflammation development primarily affecting behavior-relevant brain regions. To further study the role of GHSR in glial cells, GHSR-specific deletion or overexpression in microglia or astrocytes would be advantageous.

Of note, there are certain limitations of this study, and we hope to address them in future studies. Firstly, no female mice were involved in the experiments, so sexual dimorphism could not be identified in terms of the changes of signaling pathways, inflammatory state, and behaviors; Second, to compare the depressive or anxiety state, except forced swimming or open field test, more tests could support the data stronger such as the sucrose performance test or elevated plus maze; Last but not least, to dig out a deeper mechanism of the neuronal GHSR regulated emotional and cognitive behaviors, electrophysiology could be a powerful tool to investigate properties of excitatory and inhibitory postsynaptic potentials.

**Figure 9:**
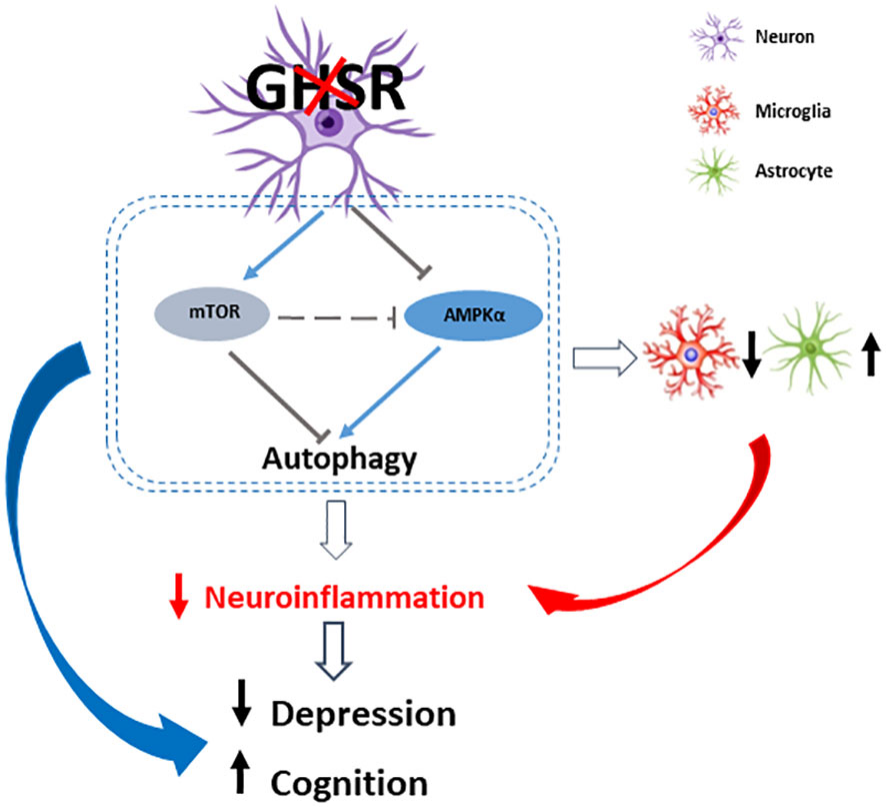
A schematic diagram of how neuronal GHSR deletion improves depression and cognition in DIO by modulating neuroinflammation through AMPK-autophagy signaling. Ablation of GHSR in neurons suppresses cortical and hippocampal inflammation, which at least in part improves DIO-associated depression and spatial learning/memory impairment. This anti-inflammatory tone is modulated by decreased AMPK signaling and inhibited autophagy signaling. Red and black arrows indicate the expression changes in neuronal GHSR deletion mice compared to WT mice: upward arrow = increase, downward arrows = decrease. The gray T bar shows the inhibitory signals; the blue line arrows show the excitatory signals.

In summary, we report for the first time that under DIO, neuron-specific GHSR deletion improved the depressive state and enhanced spatial cognitive function, showing suppression of AMPK-autophagy signaling in the hippocampus and cortex (**Figure 9**). We further demonstrated that consistent changes in the AMPK and autophagy pathways occur in GHSR-inhibited neuronal cells, indicative of the cell-autonomous effect of GHSR on these pathways in neurons. Moreover, our findings indicate that neuron-specific GHSR deletion attenuates DIO-induced neuroinflammation, as demonstrated by decreased proinflammatory chemokine and cytokine expression and altered glial cells in the cortex and hippocampus. The attenuated cortical and hippocampal inflammation likely underlies the improved emotional and cognitive functions in neuronal GHSR deficit mice. Thus, this study provides novel insight into GHSR in neurobehaviors by modulating neuron-glial interactions, which links nutrient sensing ghrelin signals to neurobehaviors. Our novel findings suggest that neuronal GHSR may be a novel therapeutic target for psychiatric and cognitive disorders.

## Data availability statement

The datasets presented in this study can be found in online repositories. The names of the repository/repositories and accession number(s) can be found in the article/**Supplementary Material**.

## Ethics statement

All animal experimental protocols were approved by the Institutional Animal Care and Use Committee of Texas A&M University (IACUC 2022-0108), and all methods were performedin accordance with the relevant guidelines and regulations.

## Author contributions

HW: Conceptualization, Investigation, Writing – review & editing, Data curation, Formal Analysis, Methodology, Software, Validation, Writing – original draft. ZS: Conceptualization, Formal Analysis, Investigation, Writing – original draft, Writing – review & editing. C-SW: Conceptualization, Writing – review & editing, Methodology, Supervision. PJ: Writing – review & editing, Investigation. JN: Writing – review & editing. CG: Writing – review & editing. SK: Writing – review & editing. DT: Writing – review & editing. JL: Writing – review & editing. YZ: Writing – review & editing. XX: Writing – review & editing, Supervision. HZ: Writing – review & editing. YS: Writing – review & editing, Conceptualization, Funding acquisition, Investigation, Resources, Supervision.
