# Supplementary material for: Neuronal ablation of GHSR mitigates diet-induced depression and memory impairment via AMPK-autophagy signaling-mediated inflammation
Source: Front Immunol. 2024 Feb 23;15:1339937. doi: 10.3389/fimmu.2024.1339937 (PMC10920242; doi:10.3389/fimmu.2024.1339937)
Supplement: Supplementary file 1 [file DataSheet_1.pdf]

Supplementary Material

1 Supplementary Figure

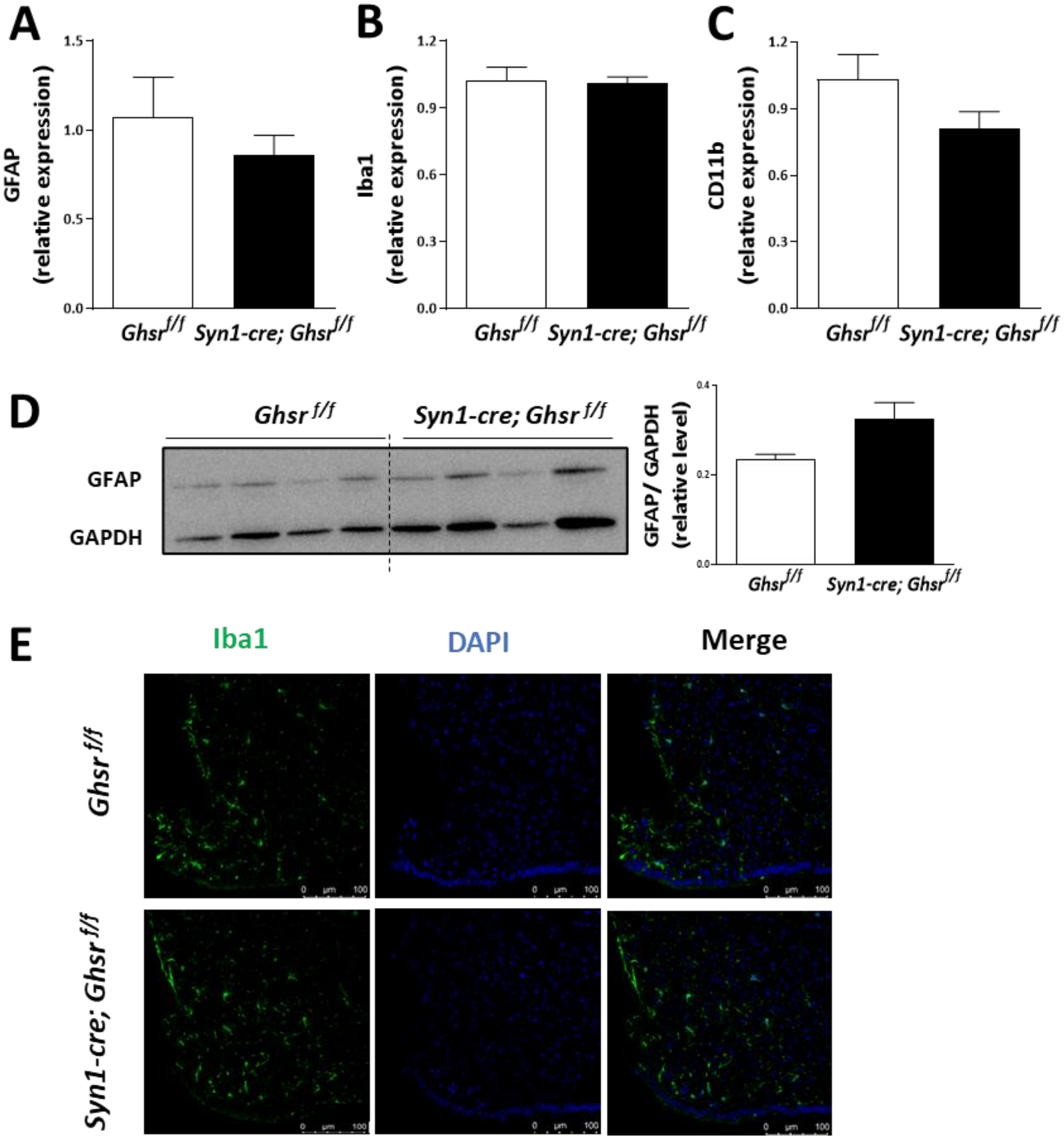

Supplementary Fig. Astrocyte and microglial characterization in the hypothalamus. The HFD was started at 2 months of age, and all mice were sacrificed at 9-10 months of age. (**A**-**C**) Gene expression of GFAP, Iba1 and CD11b. (**D**) Representative immunoblots of GFAP expression and quantification. Total protein (30 µg) was loaded. (**E**) Representative coronal sections of Iba1 expression in the hypothalamus. Iba1-labeled microglia (green), and nuclear counterstain (DAPI, blue). Scale bar 100 µm. Under a 20x objective. Mouse number n=4-5. All data are presented as the means ± SEMs.
